# Correction to “Sweet and Blind Spots in E3 Ligase Ligand Space Revealed by a Thermophoresis-Based Assay”

**DOI:** 10.1021/acsmedchemlett.1c00693

**Published:** 2021-12-28

**Authors:** Samuel Maiwald, Christopher Heim, Birte Hernandez Alvarez, Marcus D. Hartmann

Regrettably, we noticed a mistake in the calculation of the IC_50_ (and hence the *K*_i_) values for avadomide, iberdomide, lenalidomide, and pomalidomide. These were indicated in Figure 5, which also had the chemical structures of lenalidomide and pomalidomide reversed. In the main text, it should read that iberdomide has the highest affinity, pomalidomide the second highest affinity, and dasabuvir the third highest affinity of the hTBD binders tested in this study. Apart from this change in the ranking, the conclusions of the study remain valid. A corrected version of Figure 5 is provided below; the correct values are as follows: avadomide: IC_50_ = 19.4 ± 1.8 μM, *K*_i_ = 6.66 ± 0.94 μM; iberdomide: IC_50_ = 8.02 ± 0.60 μM, *K*_i_ = 0.765 ± 0.31 μM; lenalidomide: IC_50_ = 18.9 ± 1.5 μM, *K*_i_ = 6.40 ± 0.8 μM; pomalidomide: IC_50_ = 12.9 ± 2.7 μM, *K*_i_ = 3.28 ± 1.4 μM. In addition, Figure S1 and Figure S3 are interchanged in the Supporting Information: Figure S1 shows “Influence of initial fluorescence on MST behavior”, while Figure S3 shows “FRET assay data for avadomide, nitrofurantoin and UMP”.

**Figure 5:**
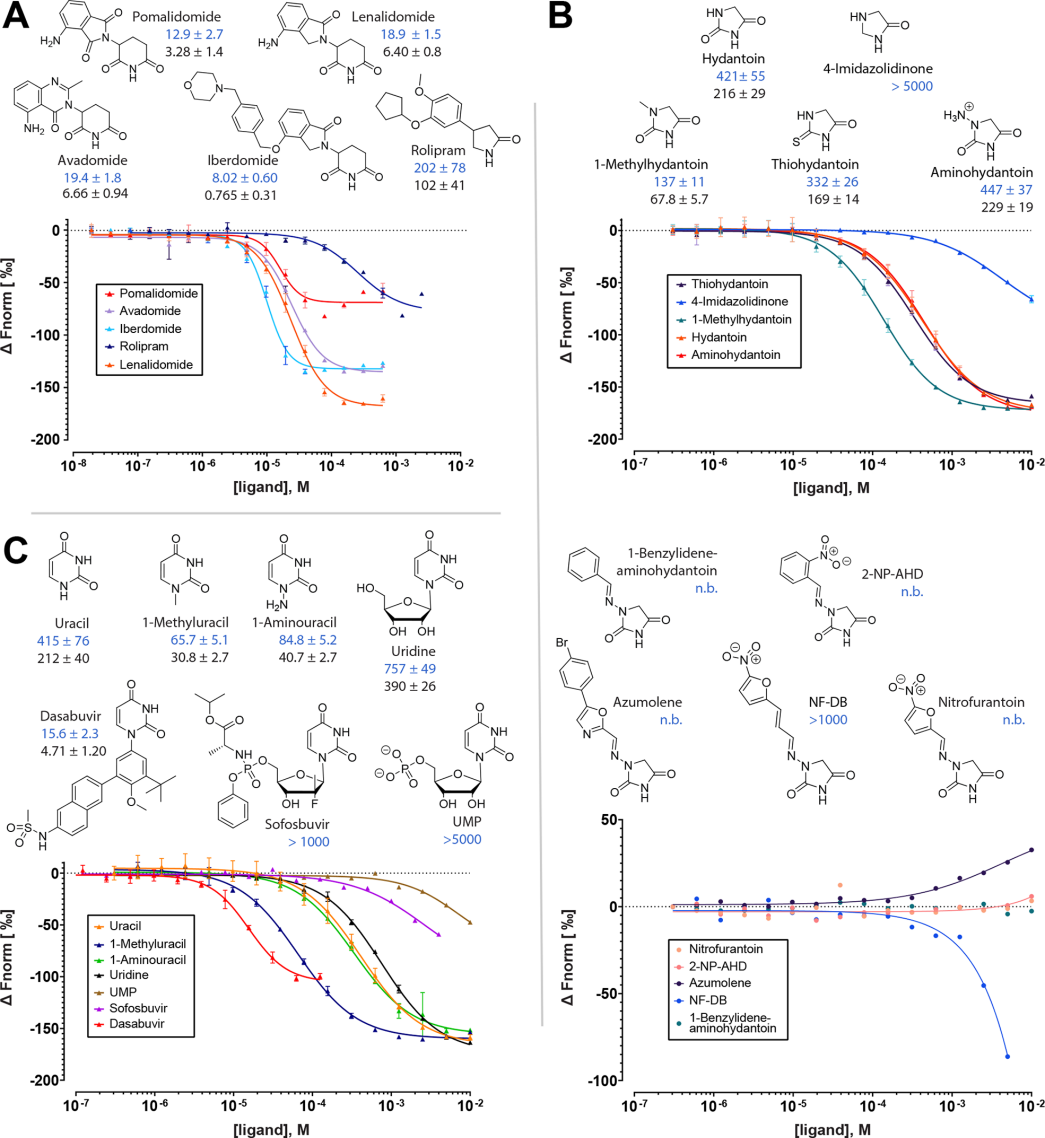
Chemical structures, dose–response curves and affinity values for (A) IMiDs and rolipram, (B) small hydantoins and hydantoins branched via hydrazo groups, and (C) uracils to hTBD. IC_50_ and derived *K*_i_ values are shown in blue and black, respectively, together with their standard deviations. All values are in μM. n.b., no binding. 2-NP-AHD, 1-(2-nitrobenzylideneamino)hydantoin; NF-DB, 1-(3-(5-nitrofuran-2-yl)allylidene)amino)hydantoin.

